# Long noncoding RNA MIR99AHG promotes gastric cancer progression by inducing EMT and inhibiting apoptosis via miR577/FOXP1 axis

**DOI:** 10.1186/s12935-020-01510-6

**Published:** 2020-08-27

**Authors:** Qingyang Meng, Xiangjun Wang, Tongqing Xue, Qiangfang Zhao, Wei Wang, Kun Zhao

**Affiliations:** 1 Department of General Surgery, Zhongshan Hospital, Fudan University, Shanghai, China; 2 Department of General Surgery, The Affiliated Hospital of Yangzhou University, Yangzhou University, Yangzhou, China; 3 Department of Oncology, Huaian Hospital of Huaian City, No.161 Zhenhuailou East Road, Huai’an, 223200 Jiangsu China

**Keywords:** Gastric cancer, MIR99AHG, miR-577, lncRNA, ceRNA, miRNA, FOXP1

## Abstract

**Background:**

Long non-coding RNAs (lncRNAs) play a vital role in the genesis and development of human cancer. LncRNA MIR99AHG has been reported to be upregulated in acute myeloid leukemia (AML); however, its function in gastric cancer (GC) is still not clear. Here we were aiming to screen the prognostic lncRNA candidates and to explore the function of MIR99AHG in GC.

**Methods:**

We have preliminarily screened some candidate lncRNA biomarkers in GC tissues through analyzing microarray datasets. The expression level of MIR99AHG in GC cell lines and tissues was monitored via qPCR. Survival analysis was performed with the patients of our hospital and TCGA database cases. CCK-8 assay, trans-well assay and flow cytometry were performed to determine cell proliferation, invasion, migration and apoptosis. Meanwhile, a target of MIR99AHG was predicted and identified by luciferase reporter gene detection experiments.

**Results:**

MIR99AHG was strongly up-regulated in human GC and contributed to cancer progression. Kaplan–Meier analysis revealed that up-regulating MIR99AHG expression was positively correlated with unfavorable overall survival (P < 0.01) of patients from our hospital and TCGA database. Knockdown of MIR99AHG expression inhibited cell proliferation, invasion, migration and promoted cell apoptosis. Moreover, MIR99AHG worked as an oncogenic gene though competing for endogenous RNA (ceRNA) of miR-577.

**Conclusions:**

Our findings suggested that MIR99AHG contributes to malignant phenotypes of GC and may become a promising therapeutic target.

## Background

Gastric cancer (GC) is the leading cause of cancer-related mortality worldwide [1]. The genesis and progression of GC is an intricate process involving numerous coding and non-coding genes [2]. Surgery is still the optimal treatment for GC patients. The survival of GC patients has been improved with the development of early detection and radical surgery [3]. However, the prognosis of GC patients remains not optimistic despite advances in surgical techniques, chemotherapy, radiotherapy and targeted therapy [4]. Therefore, identifying valuable therapeutic targets is extremely important for the GC treatment [5].

Long non-coding RNAs (lncRNAs) are characterized as transcripts of over 200 nucleotides in length, which have been demonstrated to play pivotal roles in cancer development [6]. It is known that lncRNAs were widely transcribed in the genome, but our understanding of their functions was limited [7]. Notably, accumulative evidence disclosed the indispensable involvement of lncRNAs in human malignancies in either pro-tumoral or anti-tumor manners [8].

The MIR99AHG is located on chromosome 21q21.1 and transcribed as a polycistronic primary transcript that produces a spliced lncRNA and three intronic microRNAs (miRNAs): MIR99A, MIR125B2, and LET7C (MIRLET7C). The lncRNA MIR99AHG has a role in cell proliferation and differentiation. Emmrich et al. reported that MIR99AHG, which they called MONC, showed significantly higher expression in acute megakaryoblastic leukemia (AMKL) cell lines. The knockdown of MIR99AHG impeded the proliferation of AMKL cell lines and patient-derived samples [9]. However, whether this lncRNA correlates with GC is currently unknown; therefore, here we first analyzed MIR99AHG expression levels in GC tissues and evaluated the relationship between the expression levels and the clinicopathological features of GC patients. Then experiments were carried out to detect the function of MIR99AHG in GC and the underlying mechanisms.

## Methods

### Bioinformatical analysis

The human microarray datasets (GSE109476) were downloaded from Gene Expression Omnibus (GEO) database and background adjusted by using Robust Multichip Average. Limma package was applied to analyze microarrays for gene expressions of lncRNAs. The Cancer Genome Atlas (TCGA) data were downloaded from the GDC portal. EdgeR package was applied to analyze TCGA data on a local computer for gene expression.

### Tissue samples and clinical data collection

In this research, we analyzed 118 patients who underwent resection of the primary GC at the Zhongshan Hospital, Fudan University, and Shanghai, China. This study was approved by the Ethics Committee on Human Research of the Zhongshan Hospital, Fudan University, Shanghai, China and all the patients have signed out the written informed consent. The clinicopathological information of all the patients was listed in Table 1. All the patients have been undergoing follow-up until November 2018, and the median follow-up period was 36 months (range 20–48 months). OS was defined as the interval between the surgery time and death time. The specimens were treated following the ethical standards. This study was conducted in accordance with the Declaration of Helsinki.

**Table 1:** Correlation between MIR99AHG expression and clinicopathological characteristics of GC

Clinical parameter	MIR99AHG	Expression	P-value
	High no. cases (n = 59)	Low no. cases (n = 59)	
Age (years)			0.732
≤ 50	31	36	
> 50	28	23	
Gender			0.554
Male	37	33	
Female	22	27	
Location			0.679
Distal	20	24	
Middle	21	23	
Proximal	18	12	
Size (cm)			0.654
> 5	35	33	
≤ 5	29	31	
Histologic differentiation			0.008
Well	9	13	
Moderately	17	20	
Poorly	21	10	
Undifferentiated	12	16	
Invasion depth			< 0.001
T1	4	15	
T2	10	21	
T3	20	15	
T4	15	8	
TNM stages			0.003
I–II	20	35	
III–IV	39	24	
Lymphatic metastasis			< 0.001
Yes	39	23	
No	20	36	
Distant metastasis			0.02
Yes	15	7	
No	44	52	

### Cell culture

Human GC cell lines SGC7901, BGC823, MGC803, AGS and MKN45 and the normal gastric epithelium cell line (GES-1) were purchased from the Chinese Academy of Sciences Committee (Shanghai, China). MGC803, AGS and BGC823 cells were cultured in RPMI 1640; MKN45, GES-1 and SGC7901 cells were cultured in DMEM (GIBCO-BRL) medium supplemented with 10% fetal bovine serum (FBS; Gibco Life Technologies, Gaithersburg, MD), 1% penicillin and streptomycin (Gibco Life Technologies). The cells were cultured at 37 °C in 5% CO_2_.

### RNA preparation and quantitative real-time PCR

Total RNA was extracted from cells or tissues with TRIzol reagent (Invitrogen, USA) following the protocol. RNA was qualified using a NanoDrop spectrophotometer and the A260/A280 ratio was used to test the RNA purity. Total RNA was converted to cDNA by reverse transcription and then quantitative real-time PCR (qRT-PCR) was carried out using the commercial kit (Invitrogen, USA). Relative transcript expression levels were calculated by the comparative 2^−ΔΔCT^ method normalize to GAPDH.

### CCK-8 assay

Cell Counting Kit-8 (Sigma, Japan) was applied to determine the cell viability of GC cells. Cells were seeded in 96-well plates at 1 × 10^4^ cells per well and cultured for 24 h. 10 μL of the cell proliferation reagent CCK-8 to each well and mixed then incubated for 90 min at 37 ℃. The optical density (OD) of each well was detected at wavelength of a 450 nm.

### Cell migration and invasion assays

Cell migration and invasion assay were carried out using transwell chambers (8-μm pore size; Millipore, Bedford, MA, USA) coated without/with Matrigel (Sigma-Aldrich, St. Louis, MO, USA). After transfecting for 48 h, cells suspended in serum-free media were seeded in the upper transwell chamber. The lower chamber was then added with medium containing 10% FBS. After incubation for 24 h, cells had migrated or invaded through the membrane were fixed with formaldehyde and stained with 0.1% crystal violet after wiping out cells remaining on the upper membrane. Then cells were imaged and counted with the microscope. Independent experiments were carried out for 3 times.

### Western blotting analysis

Cells were lysed with RIPA lysis buffer and total protein was extracted and quantified with the BCA kit (Beyotime, Beijing, China). The sodium dodecyl sulfate-polyacrylamide gel electrophoresis (SDS-PAGE) was used to separate 20 µg of protein extract and polyvinylidene fluoride (PVDF) membrane (Millipore) was used for transferring the protein from the gel. GAPDH was applied for normalization.

### Flow-cytometric analysis

Cells were double-stained with fluorescein isothiocyanate (FITC)-Annexin V and propidium iodide was done by the FITC Annexin V Apoptosis Detection Kit (Nanjing KeyGen Biotech. Co. Ltd., China). Following the manufacturer’s protocol. Cell apoptosis was analyzed by using Cell Quest software on a FACSAria Flow Cytometer (BD Inc., USA). Fluorescence was detected with an excitation wavelength of 480 nm. Cells were analyzed with FACSAria Flow Cytometer (BD Biosciences, USA).

### Gene transfection

Overexpressed plasmids and siRNAs together with the negative controls (GenePharma, China) were transfected into cells according to the manufacturer’s protocol of the Lopfectamine 2000 (Invitrogen, USA).

### Dual-luciferase reporter assay

The mutant type and wild type of luciferase reporter vector targeting MIR99AHG or FOXP1 binding sites were synthesized (Promega, USA). Luciferase activities were detected after transfection for 48 h. Renilla luciferase activity was used for normalization.

### Statistical analysis

All statistical analyses were carried out with SPSS v.20.0 (SPSS) and GraphPad Prism 7.0. Experiments were repeated for at least 3 times and Student’s -test and one-way ANOVA were mostly performed. Survival curves were plotted using the Kaplan–Meier method and with log-rank tests comparison, Survival data were analyzed with univariate and multivariate Cox proportional hazards models. Two-tailed P values were applied with a value of P < 0.05 as significant results.

## Results

### MIR99AHG upregulation was associated with aggressive clinical characteristics and unfavorable prognosis of GC

**Fig. 1:**
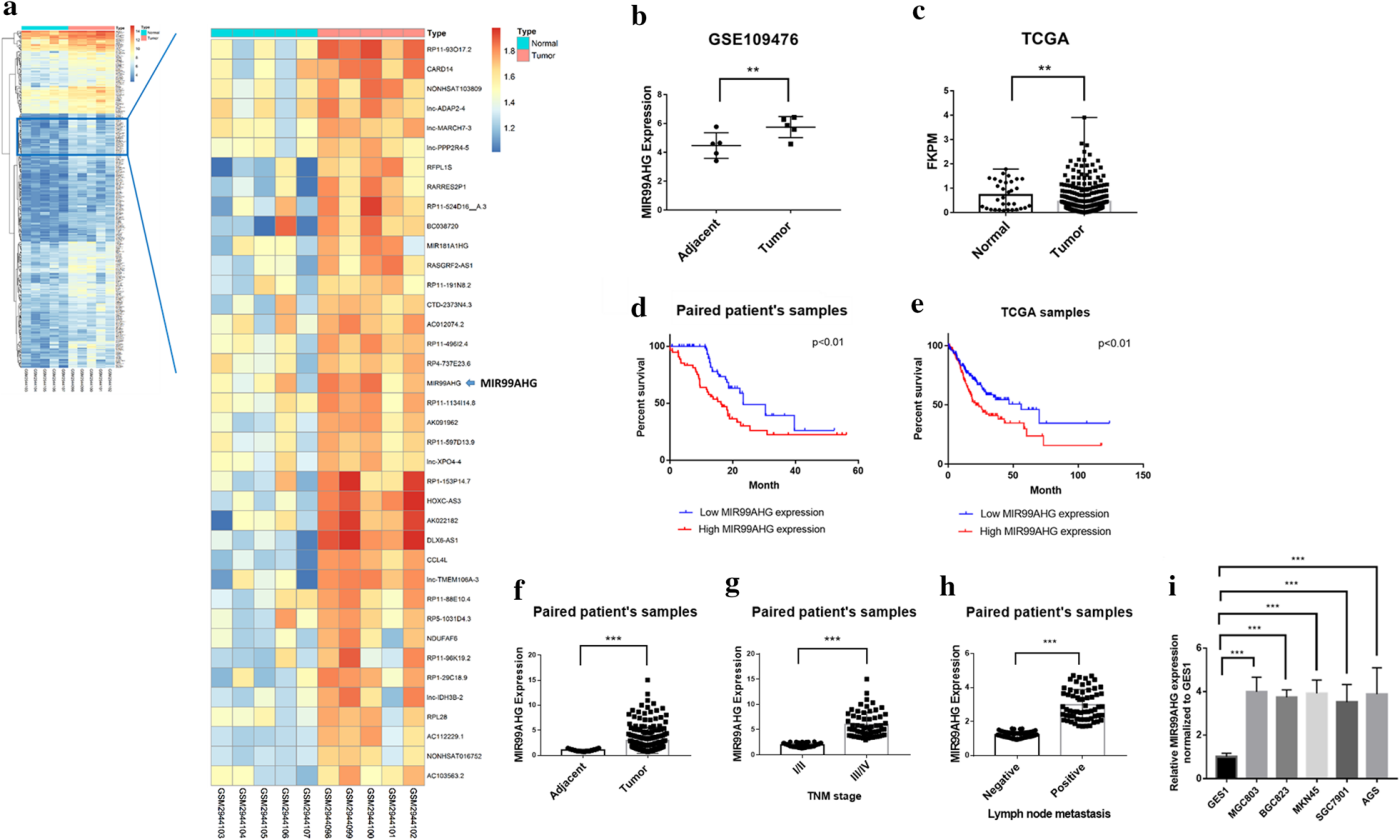
MIR99AHG upregulation was associated with aggressive clinicopathological traits and poor prognosis for GC. **a** Heatmap of up-regulated lncRNAs between GC tissues and adjacent normal tissues. The data were taken from the public microarray database. The right panel is the local zoom in on the left panel. **b** Relative expression of MIR99AHG in GC tissues and adjacent normal tissues in the GEO database. **c** Relative expression of MIR99AHG in GC tissues and normal tissues in the TCGA database. **d** Overall survival analysis of paired patient samples with GC based on MIR99AHG expression status (P < 0.01, log-rank). **e** Overall survival analysis of TCGA cases based on MIR99AHG expression status (P < 0.01, log-rank). **f** MIR99AHG expression was significantly higher in GC specimens than in the paired adjacent normal tissues. **g**, **h** MIR99AHG expression was significantly higher in patients with advanced TNM stage and lymph node metastasis. MIR99AHG expression was examined by qPCR and normalized to 18sRNA expression. **i** Relative expression of MIR99AHG in five gastric cancer cell lines (SGC7901, BGC823, MGC803, AGS and MKN45) and human normal gastric epithelial cell line (GES1) analyzed by quantitative RT-PCR. *p < 0.05, **p < 0.01 and ***p < 0.001 (two-way ANOVA)

**Fig. 2:**
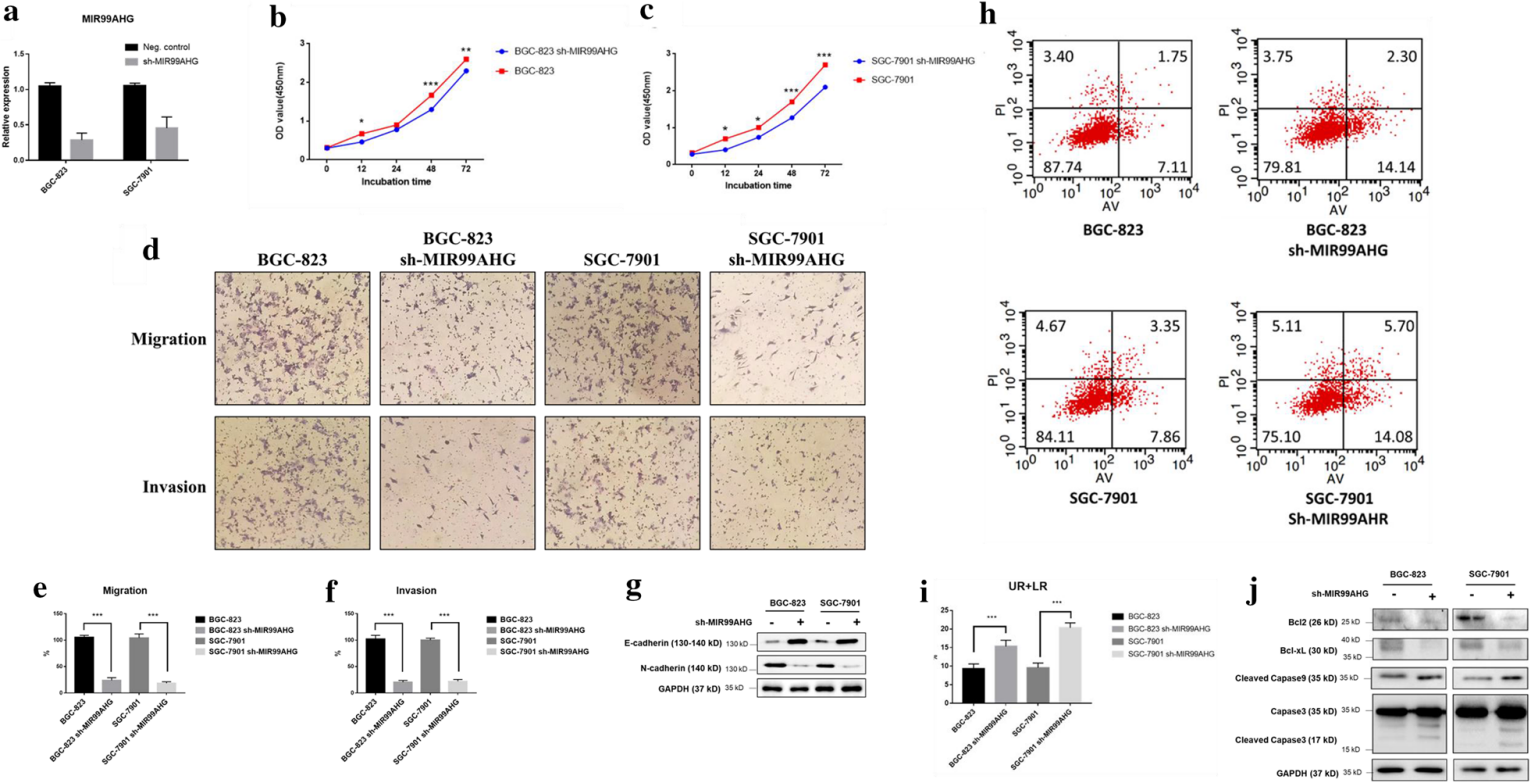
Knockdown of MIR99AHG suppressed GC cell proliferation, invasion and migration in vitro. **a** MIR99AHG expression was measured by qPCR. **b**, **c** Growth curves were measured by CCK-8 assay. **d** Trans-well assay; upper panel shows migration effect and lower panel shows invasion effect. **e**, **f** Qualification of the trans-well assay. **g** E-cadherin and N-cadherin expression levels were detected by Western blotting. **h**, **i** Knockdown MIR99AHG promoted apoptosis in GC cells. Representative FACS images and statistics based on three independent experiments. **j** Apoptosis related key proteins were detected by Western blotting. *p < 0.05, **p < 0.01 and ***p < 0.001 (two-way ANOVA)

To explore the significant lncRNAs in GC, 5 paired global expression profiles of lncRNAs in GC and adjacent tissues were downloaded from the GEO database (GSE109476). There were 355 unregulated lncRNAs showed obvious fold changes in cancer group compared to adjuvant normal tissues, logFC > 1 and padj < 0.01 (Fig. 1a). Among them, the function of MIR99AHG in GC was not defined. The median MIR99AHG expression levels in the GC tissues were 2.4 times higher than those in the adjuvant normal tissues (Fig. 1b). Then MIR99AHG expression levels were also investigated in the patients from the TCGA database, the MIR99AHG expression level was higher and showed broader ranging in GC group compared with normal group (Fig. 1c). Kaplan–Meier analysis and log-rank test were carried out to investigate the relationship between MIR99AHG expression and the clinicopathological features on overall survival (OS). Firstly, the OS was explored in the 118 paired samples, the results showed that the MIR99AHG high group had inferior overall survival (median OS: 16 months) than low group (median OS: 23 months; P < 0.01) (Fig. 1d). The 3-years OS rates were 22.4% (16.9–27.7%) in the MIR99AHG high group and 39.1% (33.9–45.9%) in the MIR99AHG low group. Univariate analyses of predictive clinical variants for survival were shown in Table 2. Then we analyzed the relevance MIR99AHG expression with OS of GC patients in 413 cases from TCGA database. Among those cases, patients were grouped into those with above-median MIR99AHG expression and below-median MIR99AHG expression. Below-median MIR99AHG expression was associated with inferior OS (median OS: 21 months) compared with the above-median (median OS: 46 months; P = 0.0144) (Fig. 1e). The 3-year OS was 41.45% (33.6–49.8%) in the above-median MIR99AHG expression group and 55.97% (47.5–62.8%) in the below-median MIR99AHG expression group. Then expression levels of MIR99AHG were determined by qPCR in 118 paired GC and adjacent non-tumor tissues. Expression levels of MIR99AHG in tumor tissues were remarkably higher than that in adjacent tissues (Fig. 2f). Further analyses of the paired patient’s samples revealed that MIR99AHG expression in GC tissues was positively related to advanced TNM stage and lymph node metastasis (Fig. 1g, h). The MIR99AHG high group (n = 59) showed poorer histologic differentiation (P = 0.008, Table 1), higher invasion depth (P < 0.001), advanced TNM stage (P = 0.003), significantly increased lymph nodes metastasis (P < 0.001) and higher distant metastasis risk (P = 0.02) than the MIR99AHG low group (n = 59). There showed no significant correlation between MIR99AHG expression and gender, age, tumor location, and tumor size (P > 0.05). In addition, qPCR assays were carried out to determine the expression of MIR99AHG in 4 GC cell lines, namely MGC803, BGC823, MKN45, SGC7901, and AGS. MIR99AHG was found to be highly expressed in all the 4 GC cell lines in comparison to GC cell lines (Fig. 1i).

**Table 2:** Univariate and multivariate Cox regression analysis for OS of patients in study cohort (n = 118)

Variables	OS		
	HR	95% CI	P value
Univariate analysis			
Age (≤ 50 years vs. > 50 years)	0.774	0.418–1.490	0.588
Gender (male vs. female)	1.265	0.891–1.608	0.346
Location (distal vs. middle + proximal)	0.910	0.408–1.469	0.577
Tumor size (> 5 cm vs. ≤ 5 cm)	1.266	0.846–1.794	0.706
Histologic differentiation (poorly + undifferentiated vs. well + moderately)	1.640	0.848–2.426	0.178
Invasion depth (T3 + T4 vs. T1 + T2)	2.118	1.146–3.312	0.014
TNM stage (III + IV vs. I + II)	3.098	1.661–4.849	0.007
Lymphatic metastasis (yes vs. no)	1.211	0.594–1.459	0.057
Distant metastasis (yes vs. no)	1.298	0.792–1.899	0.030
Expression of MIR99AHG (high vs. low)	1.473	1.287–2.054	0.001
Multivariate analysis			
TNM stage (III + IV vs. I + II)	2.699	1.401–4.984	0.006
Invasion depth (T3 + T4 vs. T1 + T2)	1.121	0.504–1.683	0.134
Lymphatic metastasis (yes vs. no)	1.073	0.691–1.662	0.455
Distant metastasis (yes vs. no)	0.998	0.701–1.492	0.074
Expression of RP11-66B24.7 (high vs. low)	2.851	1.044–4.630	0.035

### Knockdown of MIR99AHG suppressed GC cell proliferation, invasion, migration and promoted the apoptosis of GC cells in vitro

In order to explore the function of MIR99AHG in GC, we knocked down the MIR99AHG in BGC-823 and SGC-7901 cells by using the RNA interference technique (RNAi). The shRNA showed a good knockdown effect of MIR99AHG (Fig. 2a). Then CCK-8 assays were carried out to detect the role of MIR99AHG on GC cell proliferative ability. The CCK-8 results showed that knockdown of MIR99AHG reduced the cell proliferative ability of BGC-823 and SGC-7901 (Fig. 2b, c). Trans-well assay results revealed that knockdown of MIR99AHG impaired the invasion and migration of GC cells (Fig. 2d–f). Then we detected molecular markers of EMT in the protein level following MIR99AHG knockdown. The expression of E-cadherin was upregulated while N-cadherin was downregulated when MIR99AHG was knockdown (Fig. 2g). Flow cytometry analysis revealed that MIR99AHG knockdown further promoted the apoptotic rates (early + late-stage apoptosis) of GC compared with the control group (Fig. 2h, i). Western blot analysis demonstrated that MIR99AHG knockdown group showed downregulated expression of anti-apoptotic proteins Bcl-2 and Bcl-xl and upregulated expression of pro-apoptotic proteins cleaved caspase-3 and caspase-9 (Fig. 2j). These results suggested that knockdown of MIR99AHG may suppress GC cell proliferation, invasion, migration and promote the apoptosis of GC cells.

### MIR99AHG/miR-577/FOXP1 axis regulated the malignant phenotype of GC cells

**Fig. 3:**
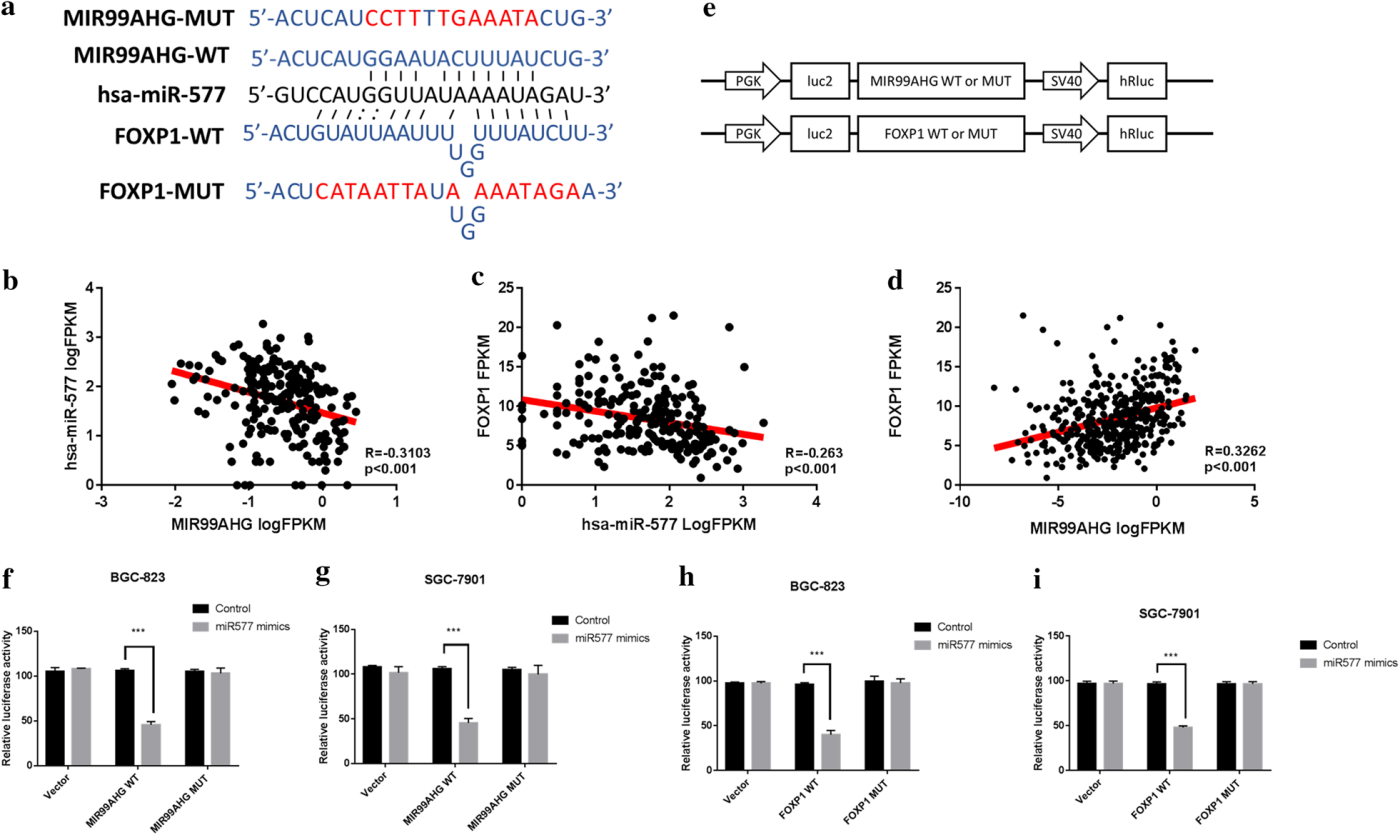
MIR99AHG functioned as a molecular sponge of miR-577, liberating FOXP1 transcripts. **a** Diagram of putative binding sits for miR-577 in MIR99AHG and FOXP1. **b**–**d** Correlation assay was performed on TCGA cases, Pearson method was used to calculate the R-value. **e** the schematic of mutant MIR99AHG and FOXP1 constructs. **f**–**i** miR-577 mimics or negative control were co-transfected into BGC-823 and SGC-7901 cells. Luciferase activity was detected 24 h after transfection using the dual-luciferase assay. *p < 0.05, **p < 0.01 and ***p < 0.001 (two-way ANOVA)

Researches have revealed that lncRNAs can act as competing endogenous RNAs (ceRNA) for miRNAs. The potential miRNAs that might be sponged by MIR99AHG were predicted through starBase tool (https://starbase.sysu.edu.cn). Then we identified MIR99AHG might sponge to miR-577. Additionally, TargetScan analysis predicted the target region for miR-577 in the 3′-UTR of the FOXP1 gene (Fig. 3a), suggesting that miR-577 may directly target FOXP1. Thus, we hypothesized that MIR99AHG might promote FOXP1 expression by interacting with miR-577. Through correlation analysis on TCGA cases, we confirmed that miR-577 expression was negatively correlated with MIR99AHG expression (Fig. 3b). In addition, FOXP1 expression was negatively regulated by miR-577 (Fig. 3c). Then, as expected, expression of MIR99AHG showed a positive correlation with FOXP1 (Fig. 3d). To further validate miR-577 directly binding to FOXP1, the dual-luciferase assay was conducted in GC cell lines. Luciferase vectors were constructed as shown in the scheme (Fig. 3e). We co-transfected the miR-577 mimics and wild type MIR99AHG vector or mutant MIR99AHG vector into GC cells. Results showed that wild type MIR99AHG vector co-transfected cells exhibited lower luciferase activity (Fig. 3f, g). Consistently, miR-577 remarkably decreased the luciferase activity of wild type FOXP1 reporter but not mutated vector (Fig. 3h, i).

### MIR99AHG/miR-577/FOXP1 axis regulated the malignant phenotype of GC cells

**Fig. 4:**
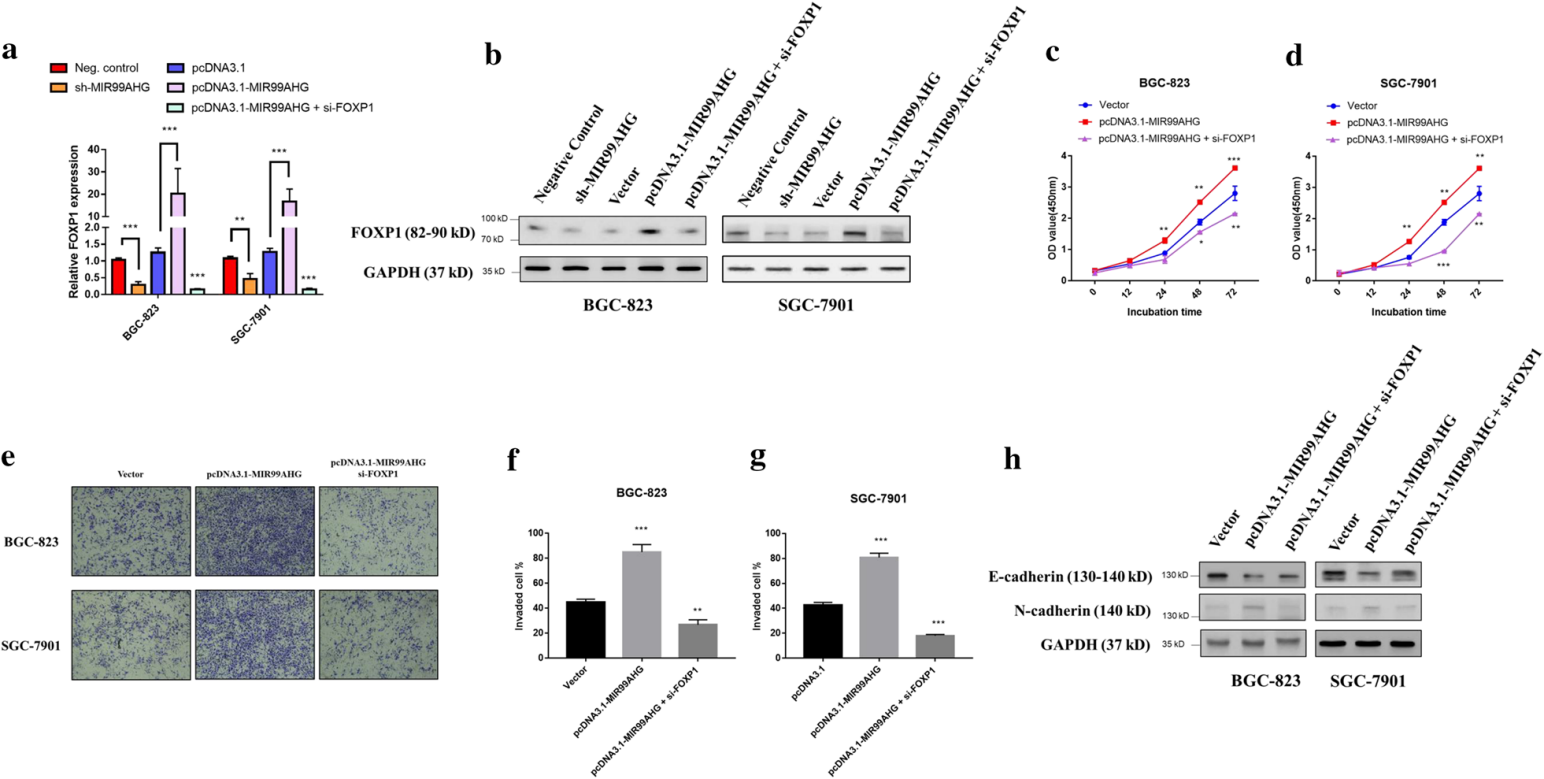
MIR99AHG/miR-577/FOXP1 axis regulated the malignant phenotype of GC cells. **a** mRNA level of FOXP1 was confirmed by qPCR in BGC-823 and SGC-7901 cell following MIR99AHG knockdown, MIR99AHG overexpression, and MIR99AHG overexpression plus FOXP1 knockdown. **b** Protein expression level of FOXP1 was confirmed by western blot in BGC-823 and SGC-7901 cell following MIR99AHG knockdown, MIR99AHG overexpression, and MIR99AHG overexpression plus FOXP1 knockdown. **c**, **d** CCK8 proliferation assay in BGC-823 and SGC-7901 cells after MIR99AHG overexpression and MIR99AHG overexpression plus FOXP1 knockdown. **e** Trans-well assay shows invasion effect. **f**, **g** Qualification of the trans-well assay. **h** E-cadherin and N-cadherin expression levels were detected by Western blotting. *p < 0.05, **p < 0.01 and ***p < 0.001 (two-way ANOVA)

Knockdown of MIR99AHG markedly suppressed FOXP1 expression in both BGC-823 and SGC-7901 cells, whereas overexpression of MIR99AHG significantly upregulated FOXP1 expression levels, in both mRNA and protein levels (Fig. 4a, b). In addition, as expected directly FOXP1 knockdown or miR-577 mimics transfection significantly decreased FOXP1 expression (Fig. 4a, b). MIR99AHG overexpression significantly promoted the proliferation and invasion of GC cells, while this effect was abolished by knockdown of FOXP1 (Fig. 4c–g). Western blot results showed that MIR99AHG overexpression significantly downregulated E-cadherin expression and upregulated N-cadherin expression, however knockdown FOXP1 partially reversed this effect (Fig. 4h). These results indicated that FOXP1 could also be regulated by MIR99AHG. MIR99AHG/miR-577/FOXP1 axis regulated migration and invasion ability of GC cells.

### FOXP1 potentiated Wnt/β-catenin signaling

**Fig. 5:**
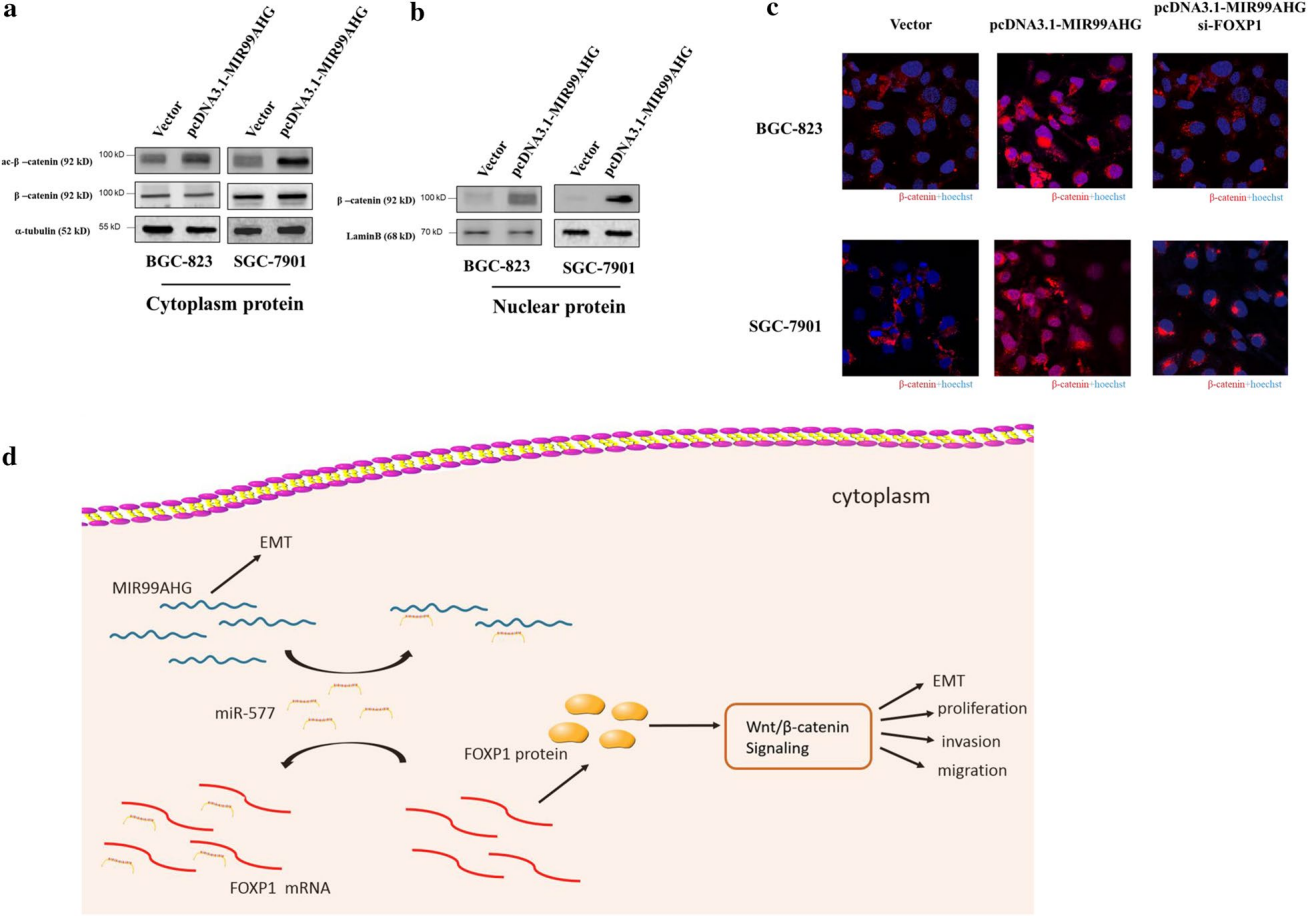
FOXP1 potentiated Wnt/β-catenin signaling. **a** Acetyl-β-catenin was detected by western blotting following MIR99AHG overexpression or MIR99AHG overexpression and FOXP1 knockdown. **b** Nuclear protein β-catenin was detected by western blotting following MIR99AHG overexpression or MIR99AHG overexpression and FOXP1 siRNA transfected 48 h. **c** ICC staining of β-catenin and Hoechst after MIR99AHG overexpression and FOXP1 siRNA transfected 48 h. **d** Schematic diagram of MIR99AHG based regulatory mechanism in GC cells

It has been reported that FOXP1 overexpression potentiated Wnt/β-catenin signaling in diverse cancer cell types [10]. FOXP1 co-complexed with a β-catenin transcriptional complex on chromatin promoted CBP [CREB (adenosine 3′,5′-monophosphate response element-binding protein)-binding protein]-dependent acetylation of β-catenin, resulting in enhanced β-catenin-dependent transcription [10, 11]. Therefore, we tested whether MIR99AHG promotes FOXP1 meditated acetylation of β-catenin at Lys49. Indeed, MIR99AHG overexpression induced acetylation of Lys49 in β-catenin and the acetylation was suppressed by siRNA-mediated silencing of FOXP1 (Fig. 5a). In addition, overexpression of MIR99AHG promoted β-catenin nuclear transportation, and the MIR99AHG mediated β-catenin nuclear transportation abolished by FOXP1 knockdown (Fig. 5b). The immunocytochemistry (ICC) results showed that MIR99AHG overexpression stabilized β-catenin and the stabilized β-catenin mainly located in the nuclear, however the stabilization phenomena was abolished by FOXP1 knockdown (Fig. 5c).

## Discussion

Gastric cancer is the fifth most common malignancy with a third highest cancer associated death rate [12]. Recently, various studies have suggested that lncRNAs are closely related to the carcinogenesis and development of GC [13–17]. LncRNA TINCR could promote the metastatic ability of GC cells by regulating the stability of KLF2 [18, 19]. LncRNA SNHG16 was reported to promote the proliferation, invasion and apoptosis of GC by sponging miR-135, thus activating JAK2/STAT3 pathway [20]. Moreover, lncRNA MALAT1 and MACC1-AS1was demonstrated to regulate the chemoresistance of GC [16, 21]. Therefore, exploring the roles and mechanisms of lncRNAs might shed new light on the diagnosis and treatment of GC [22, 23].

Here we elucidated the aberrant upregulation of MIR99AHG in GC. Firstly, we downloaded and analyzed previous microarray datasets (GSE109476) to explore the different lncRNA expression patterns between cancer and adjuvant normal tissues. We found that MIR99AHG was significantly upregulated in GC cancer tissues. To further investigate the expression difference, we compared MIR99AHG expression levels in the patients from the TCGA database. Similarly, MIR99AHG expression levels were higher and showed broader ranging in GC group compared with normal group. Later, we investigated 118 paired GC cancer tissues and non-cancer tissues. The survival curve revealed that the MIR99AHG level was negatively related to the OS of GC. The correlation between MIR99AHG expression and clinicopathological features of GC patients was further explored. The results showed that expression of MIR99AHG was positively associated to TNM stage and lymph node metastasis (Table 2). Subsequent experiments showed that knockdown of MIR99AHG suppressed the proliferation and invasion of the GC cells by regulating EMT and meanwhile, induced apoptosis progression.

One of the important roles of lncRNAs is to act as ceRNAs by sponging to miRNAs. For example, lncRNA MIR99AHG promoted apoptosis of neuroblastoma cells by targeting miR-296-5p [24]. FOXP1, fork head box protein 1, has been demonstrated to act as a B cell oncogene and was involved in B-cell differentiation and survival [25, 26]. FOXP1 was positively associated with the expression of BCL2 and might affect cell apoptosis [27, 28]. Based on our analysis, FOXP1 expression was negatively regulated by miR-577 while MIR99AHG showed a positive relation with FOXP1. Further experiments then showed that knockdown of FOXP1 or miR-577 mimics transfection could abolish the effect of MIR99AHG on cell proliferation and migration. FOXP1 co-complexed with a β-catenin transcriptional complex on chromatin promoted CBP-dependent acetylation of β-catenin, resulting in enhanced β-catenin-dependent transcription. Our results depicted that MIR99AHG overexpression stabilized β-catenin and the stabilized β-catenin mainly located in the nuclear, however the stabilization phenomena was abolished by FOXP1 knockdown. Take together, our data suggested that MIR99AHG overexpression could sponge to miR-577, which led to an increase of FOXP1 expression. Then, overexpressed FOXP1 caused β-catenin abnormal acetylation and resulted in WNT signaling abnormal activation.

### Conclusions

Taken together, we illustrated that lncRNA MIR99AHG was highly expressed in both GC tissues and cell lines. MIR99AHG could act as a ceRNA for miR-577, thus activating the FOXP1 mediated Wnt/β-catenin pathway. Moreover, a higher level of MIR99AHG was correlated with more advanced tumor progression and poorer prognosis. Our data that MIR99AHG might be useful as a diagnostic and prognostic biomarker as well as a therapeutic target for gastric cancer in the future.

## Data Availability

Data and materials supporting the current study are all available from the corresponding author on reasonable request.

## Abbreviations

AMKL: Acute megakaryoblastic leukemia
AML: Acute myeloid leukemia
ceRNA: Competing for endogenous RNA
FBS: Fetal bovine serum
FITC: Fluorescein isothiocyanate
GC: Gastric cancer
GEO: Gene Expression Omnibus
lncRNAs: Long non-coding RNAs
miRNAs: Acute megakaryoblastic leukemia
OD: Optical density
PVDF: Polyvinylidene fluoride
qRT-PCR: Quantitative real-time PCR
SDS-PAGE: Sodium dodecyl sulfate-polyacrylamide gel electrophoresis
TCGA: The Cancer Genome Atlas
